# In‐Vitro Evaluation of HIV/SARS‐CoV‐2 Co‐Infection Mediated Proteomic Changes in Astrocytes and Pericytes Reveals Altered Signaling Pathways Associated With Neurodegenerative Disorders

**DOI:** 10.1002/jmv.71086

**Published:** 2026-08-06

**Authors:** Arpan Acharya, Michellie Thurman, Debapriya Sutar, Oluwatayo Israel Olasunkanmi, Johid R. Malik, Shetty Ravi Dyavar, Ákos Végvári, Siddappa N. Byrareddy

**Affiliations:** ^1^ Department of Pharmacology and Experimental Neuroscience, College of Medicine University of Nebraska Medical Center Omaha Nebraska USA; ^2^ University of Nebraska Medical Center (UNMC) Center for Drug Discovery, UNMC Omaha Nebraska USA; ^3^ Division of Molecular Metabolism Department of Medical Biochemistry & Biophysics (MBB) Karolinska Institutet Stockholm Sweden

**Keywords:** ACE‐2, and pericytes, astrocytes, co‐infection, COVID‐19, HIV, long‐covid, microglia, neuroHIV, PASC, SARS‐CoV‐2

## Abstract

Coronavirus disease 2019 (COVID‐19) survivors frequently experience a wide range of symptoms known as post‐acute sequelae of SARS‐CoV‐2 (PASC) or long COVID. Importantly, complications arising from microvascular dysfunction, blood‐brain barrier (BBB) disruption, and chronic neuroinflammation have been implicated in driving PASC within the central nervous system (CNS), known as neuro‐PASC. Notably, people with HIV (PWH), who suffer from chronic neuroinflammation, BBB impairment, and glial cell dysfunction, collectively known as neuro‐HIV, are generally at higher risk of neuro‐PASC. The overlap between neuro‐PASC and neuro‐HIV raises concerns that HIV and SARS‐CoV‐2 co‐infection may exacerbate neurological dysfunctions among PWH. In this study, using an in‐vitro cell culture model, we examine the effects of HIV and SARS‐CoV‐2 mono‐ and co‐infection in microglia, astrocytes, and pericytes. Our results demonstrated that majority of brain cell types support SARS‐CoV‐2 replication, in the presence and absence of HIV infection. Furthermore, in both mono‐ and co‐infected cells, there were varying degree of up‐ and downregulation of SARS‐CoV‐2 host cell entry factors, such as ACE2, TMPRSS2, NRP1, and TRIM28, and inflammatory cytokines including IL‐6, TNF‐α, and IL‐1β. Moreover, conditioned media collected from HIV, SARS‐CoV‐2, and HIV/SARS‐CoV‐2 co‐infected astrocytes and pericytes were shown to be neurotoxic. Additionally, proteomic analysis has revealed a unique set of proteins significantly up/down regulated in HIV/SARS‐CoV‐2 co‐infected astrocytes and pericytes. The gene set enrichment analysis of these proteins indicates dysregulation of lipid, energy, and immune metabolism pathways linked to neurodegenerative disorders like Alzheimer's, Parkinson's, Huntington's disease, and amyotrophic lateral sclerosis. These in‐vitro findings indicate that astrocytes and pericytes from HIV/SARS‐CoV‐2 co‐infection exhibit altered protein expression profiles, implicating dysregulated signaling pathways associated with neurodegenerative dysfunction.

**Abbreviations**
ACE2: Angiotensin converting enzyme 2
AD: Alzheimer's Disease
ALS: Amyotrophic lateral sclerosis
Aβ: Amyloid‐β
BBB: Blood‐Brain Barrier
CNS: Central nervous system
COVID‐19: Coronavirus disease of 2019
EMEM: Eagle's Minimum Essential Medium
HAND: HIV‐Associated Neurocognitive Disorder
HD: Huntington Disease
HIV: Human immunodeficiency virus
IL‐1β: Interleukin 1 beta
IL‐18: Interleukin 18
IL‐6: Interleukin 6
MDM: Monocyte‐derived macrophages
MERS‐CoV: Middle East respiratory syndrome coronavirus
MOI: Multiplicity of infection
NRP1: Neuropilin 1
PASC: Post‐acute sequelae of COVID‐19
PD: Parkinson's Disease
PNS: Peripheral nervous system
PWH: People living with HIV
SARS‐CoV: Severe acute respiratory syndrome coronavirus
SARS‐CoV‐2: Severe acute respiratory syndrome coronavirus 2
snRNA‐seq: Single‐nucleus RNA sequencing
TMPRSS2: Transmembrane serine protease 2
TNF‐α: Tumor necrosis factor alpha
TRIM28: Tripartite motif containing 28

## 1 Introduction

Coronavirus disease 2019 (COVID‐19), caused by the severe acute respiratory syndrome coronavirus 2 (SARS‐CoV‐2), first emerged in late 2019 and rapidly developed into a pandemic with severe consequences for health care systems and societies worldwide [1, 2, 3]. The impact of COVID‐19 on the central and peripheral nervous system (CNS/PNS) is well established [4, 5, 6, 7]. Notably, early manifestation of neurological symptoms has been linked to severe clinical outcomes, often associated with higher mortality and morbidity rates [8]. Moreover, the occurrence of cerebrovascular complications has also been widely observed among COVID‐19 patients with comorbidities such as aging, hypertension, and type 2 diabetes [9]. Cognitive impairment and other neurological complications like headache, vertigo, anosmia, tinnitus, and dysautonomia are reported in a subset of COVID‐19 patients long after recovery from the active disease, collectively called post‐acute neurologic sequelae of COVID‐19 (PASC). People living with human immunodeficiency virus (PWH) are considered a high‐risk group for SARS‐CoV‐2 infection, due to their compromised immune system and the overlapping co‐morbid conditions with COVID‐19 [10, 11]. HIV is well recognized for its ability to invade the CNS early after infection and establish viral reservoirs in the brain. HIV causes chronic neuroinflammation, blood‐brain barrier (BBB) disruption, and develops a spectrum of neurological complications like depression, mood swings, and memory loss, collectively known as neuro‐HIV [12, 13, 14]. Even in the era of combined antiretroviral therapy (ART), viral reservoirs and chronic immune activation in the CNS continue to drive neurological complications among PWH. While the neurological consequences of HIV are well documented, a critical knowledge gap remains in understanding how these pre‐existing CNS alterations might interact with, or be exacerbated by, a subsequent infection with SARS‐CoV‐2.

Interestingly, some clinical cohort studies of HIV‐1/SARS‐CoV‐2 co‐infection reported that PWH pose a higher risk of severe disease outcomes and mortality/morbidity [15], experience increased rates of hospitalization, prolonged recovery time, and a greater burden of neurological complications compared to people without HIV [16, 17, 18]. However, the short‐ and long‐term impacts of SARS‐CoV‐2 on the CNS of PWH, who already have a compromised immune system and leaky BBB, remain elusive. It remains unclear how SARS‐CoV‐2 may affect CNS cells like astrocytes and pericytes that maintain BBB integrity and contribute to neuroinflammation in PWH [19, 20].

To address this knowledge gap, we studied HIV/SARS‐CoV‐2 co‐infections in human brain derived astrocytes, pericytes, and microglia cells, as they are described as primary targets for SARS‐CoV‐2 [21, 22]. Our findings indicate that all examined brain cell types support SARS‐CoV‐2 replication. However, the cellular products released from SARS‐CoV‐2‐infected astrocytes and pericytes in the presence and absence of HIV were neurotoxic. Additionally, quantitative proteomics and subsequent gene set enrichment analysis of astrocytes and pericytes suggest dysregulation of energy metabolism pathways, adaptive immune responses, and synaptic transmissions associated with the development of neurodegenerative disorders. Overall, these findings suggest that SARS‐CoV‐2 infection in the presence or absence of HIV may imprint a proteomic signature in CNS derived astrocyte and pericytes, which can alter signaling pathways implicated in HIV associated neuropathogenesis.

## 2 Materials and Methods

### 2.1 Cells

To investigate the impact of HIV/SARS‐CoV‐2 co‐infection, primary human brain microvascular astrocytes (catalog # HMP202; Neuromics) and pericytes (Catalogue # HMP104; Neuromics), isolated from healthy human brain cortical tissue, were maintained using an astrocyte‐ and pericyte‐ growth medium (Catalogue # PGB003; Neuromics) and (Catalogue # PGB001; Neuromics) in T25 flasks, respectively. The media was changed every 2 days, and cells were sub‐cultured at a 1:2 ratio weekly to maintain optimal growth. C20 cells (immortalized microglia generated from human primary microglia) were maintained in DMEM:F12/10% FBS medium and used for co‐infection experiments. Calu‐3 cells were cultured in Eagle's Minimum Essential Medium (EMEM) (ATCC 30‐2003) containing 10% FBS and used for growing the viral stock.

### 2.2 Production and Titration of SARS‐CoV‐2 and HIV‐1_ADA_ Stocks

SARS‐CoV‐2 isolate USA‐WI1/2020 (BEI; cat# NR‐52384) was passaged in Calu‐3 cells for generating viral stock. The viral titer was determined using the plaque assay as described by us previously [23]. HIV‐1_ADA,_ a laboratory‐adapted M‐tropic HIV‐1 strain, was grown and titered by RT‐activity assay as described earlier [24].

### 2.3 HIV and SARS‐CoV‐2 Infection

**Figure 1:**
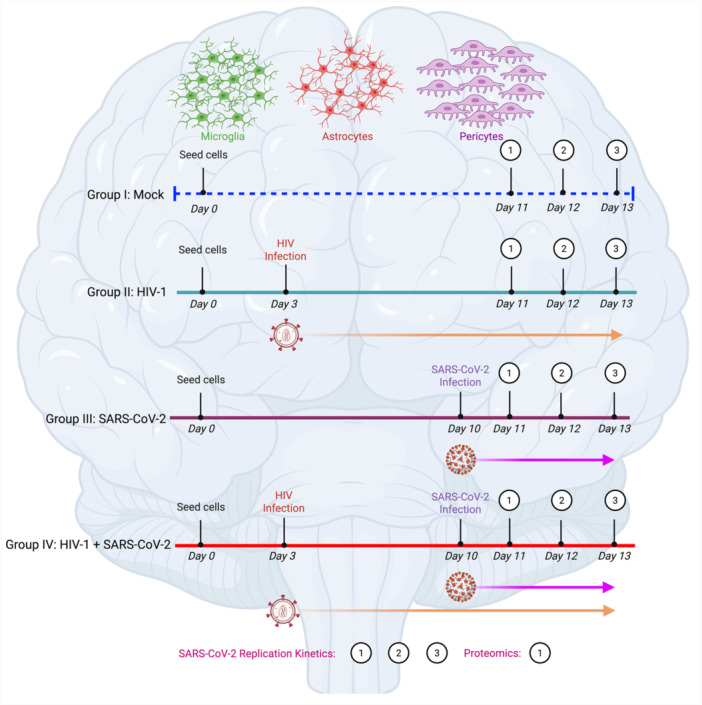
Schema representing the study design. We used human brain microvascular astrocytes and pericytes isolated from normal human brain cortical tissue, and an immortalized human microglia cell line (C20). There were four groups in the study for each cell type. Group I: Mock (uninfected controls; these cells were seeded on day 0 and harvested on day 11–13 for proteomic analysis); Group II: HIV‐1 (these cells were seeded on day 0, infected with HIV‐1 on day 3 and harvested on day 11–13 for proteomic analysis); Group III: SARS‐CoV‐2 (these cells were seeded on day 0, infected with SARS‐CoV‐2 on day 10 and harvested on day 11 for proteomic analysis and on days 11, 12, and 13 for SARS‐CoV‐2 replication kinetics evaluation; Group IV: HIV‐1+SARS‐CoV‐2 (these cells were seeded on day 0, infected with HIV‐1 on day 3, infected with SARS‐CoV‐2 on day 10, and harvested on day 11 for proteomic analysis and on days 11, 12, and 13 for SARS‐CoV‐2 replication kinetics evalution.

To examine the individual effects of HIV‐1 and SARS‐CoV‐2, as well as their co‐infection, on astrocytes, pericytes, and microglia, four experimental groups were established, as illustrated in Figure 1. Groups I consisted of uninfected controls, group II was infected with HIV‐1, group III was infected with SARS‐CoV‐2, and group IV was co‐infected with both viruses. Group II and IV cells were infected with 0.1 multiplicity of infection (MOI) of HIV‐1_ADA_ and incubated at 37°C for 4 h. Then, the cells were washed 3 times with 1X PBS, and fresh medium was added. Culture supernatants were collected at designated time points to quantify HIV‐1 RNA copies using RT‐qPCR assay [25].

Group III and IV, the cells were infected with SARS‐CoV‐2 at an MOI of 1.0, as previously described [26]. Culture supernatant was collected at 24‐, 48‐, and 72‐h post‐infection (hpi). The SARS‐CoV‐2 genomic RNA in the culture supernatant was quantified using RT‐qPCR [23].

### 2.4 Neuronal Cytotoxicity Assay

SH‐SY5Y cells were seeded in 96‐well plates in complete DMEM/F12 medium. After 24 h, the medium was replaced with Neurobasal medium supplemented with 10 µM retinoic acid (RA) and 10 µM 12‐o‐tetradecanoylphorbol‐13‐acetate (TPA). Cells were differentiated for 48 h and treated with conditioned culture supernatant collected from HIV, SARS‐CoV‐2, and HIV/SARS‐CoV‐2 co‐infected cells at a 1:10 (v/v) ratio in Neurobasal medium. After 72 h of treatment, neuronal cell viability was assessed using the MTT assay [27].

### 2.5 Quantitative Proteomics Analysis

Proteomics profiling of astrocytes and pericytes across all experimental groups was performed as previously described [28]. Briefly, at 24 h post‐infection, the cells were harvested, washed with ice‐cold 1X PBS, and proteins were extracted using an SDS‐based buffer mixed with protease and phosphatase inhibitor cocktails (Thermo Fisher SCIENTIFIC; cat # 78430). This was followed by the digestion of the proteins on S‐Trap micro columns (Protifi, USA), and then the resulting peptides were labeled with isobaric TMTpro^TM^ reagents. Next, the labeled peptides were concatenated into 12 fractions, following high pH (HpH) reversed‐phase chromatographic fractionation. Peptides in each fraction were separated by low pH reversed‐phase chromatography on an Ultimate 3000 UHPLC (Thermo Fisher Scientific, USA) in a 120 min linear organic modifier gradient. Data acquisition was performed on a Orbitrap Fusion Lumos tribrid mass spectrometer (Thermo Fisher Scientific, USA) in data‐dependent acquisition (DDA) mode, isolating the most intense precursors in 2 s at 120,000 mass resolution in the mass range of *m/z* 350 – 1500, and applying maximum injection time (IT) of 50 ms and dynamic exclusion of 45 s; precursor isolation width of 0.7 Th with high collision energy (HCD) of 34% at a resolution of 50,000 and maximum IT of 86 ms in MS2 event.

Proteins were searched against the SwissProt human databases using the search engine Sequest HT in Proteome Discoverer v2.4 (ThermoFisher Scientific, USA) software environment, allowing a maximum of two missed cleavages. Oxidation of methionine, deamidation of asparagine and glutamine, and TMTpro modification of lysine and N‐termini were set as variable modifications, while carbamidomethylation of cysteine was used as a fixed modification. The false discovery rate (FDR) was set to 1%.

### 2.6 Proteomic Data Analysis

Differential expression analysis was performed using the R package limma v3.50.0 [29]. Proteins with p.adj < 0.05 were considered significantly expressed. Pathway enrichment analysis was performed using the R package Piano v2.10.0 [30]. Pathways with p.adj(dist.dir.up) and p.adj(dist.dir.dn)< 0.1 were selected as significantly up‐regulated and down‐regulated pathways, respectively. KEGG pathway gene sets of categories, including metabolism, environmental information processing, and organismal systems, were used for the pathway enrichment analysis. Heatmaps were generated using the R package ComplexHeatmap v2.10.0 [31]. Volcano plots were created using the R package ggplot2 v3.3.5. The Venn diagram was created using the online tool InteractiVenn [32].

### 2.7 Gene Expression Analysis of SARS‐CoV‐2 Host Entry Receptors and Inflammatory Mediators in Astrocytes and Pericytes

Astrocytes and pericytes were harvested at the indicated time points (Figure 1). RNA was isolated from the cells using QIAGEN RNeasy Plus Kits (cat #74034) as described previously [33]. The messenger RNA (mRNA) was quantified using SimpliNano spectrophotometer (GE Healthcare Bio‐Sciences Corp., Piscataway, NJ, USA) and stored in a −80°C deep freezer for downstream analysis. 200 ng of RNA from each sample were converted into cDNA using SuperScript™ III First‐Strand Synthesis SuperMix (catalog#11752‐050; ThermoFisher SCIENTIFIC) following the manufacturer's instructions. The Quantitative RT‐PCR assays was performed using TaqMan® Gene Expression Assay (20X) and TaqMan™ Universal PCR Master Mix (Catalog number: 4304437). The thermal cycling conditions were set to initial denaturation at 95°C for 10 min, followed by 40 cycles of 95°C for 15 s, 60°C for 1 min. The primer/probe set used in the study was as follows: GAPDH as endogenous control (Hs02758991_g1; VIC/MGB), ACE2 (Hs01085331_m1; FAM/MGB), TMPRSS2 (Hs00237175_m1; FAM/MGB), NRP1 (Hs00826128_m1; FAM/MGB), TRIM28 (Hs00232212_m1; FAM/MGB), IL‐6 (Hs00174131_m1; FAM/MGB), TNF‐α (Hs00174128_m1; FAM/MGB), IL‐1β (Hs01555410_m1; FAM/MGB) and IL‐18 (Hs01038788_m1; FAM/MGB). The fold change in gene expression values was calculated using the 2^−Δ(ΔCt)^ method, where ΔCt = Ct_target_—Ct_housekeeping_ and Δ(ΔCT) = ΔCt infected ‐ ΔCt mock‐infected.

### 2.8 Statistical Analysis

**Figure 2:**
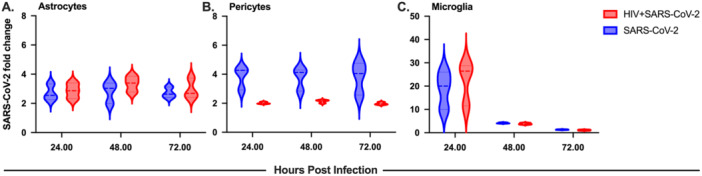
SARS‐CoV‐2 viral loads in astrocytes, pericytes, and microglia in presence and absence of HIV‐1 infection. The cells were infected with 1 MOI of SARS‐CoV‐2 and 0.1 MOI of HIV‐1 in triplicates as described in Figure 1. Culture supernatant was collected after 1 h (considered as baseline), 24 h, 48 h, and 72 h post SARS‐CoV‐2 infection. Fold change in SARS‐CoV‐2 viral loads relative to baseline at 24, 48, and 72 h post‐infection in SARS‐CoV‐2 or HIV‐1/SARS‐CoV‐2 co‐infected astrocytes (A), pericytes (B) and microglia (C). The astrocytes and pericytes from three independent donors were included in this study. All experiments were performed in triplicates and repeated three times. Microglia (C20 cells) were tested in triplicates. Multiple unpaired *t*‐tests were performed to compare the SARS‐CoV‐2 viral loads between the groups and longitudinal time points. *p*‐values were adjusted using the two‐stage step‐up method of Benjamini, Krieger and Yekutieli‐Hochberg false discovery rate (FDR) procedure and statistical significance was defined as an adjusted *p* < 0.05.

**Figure 3:**
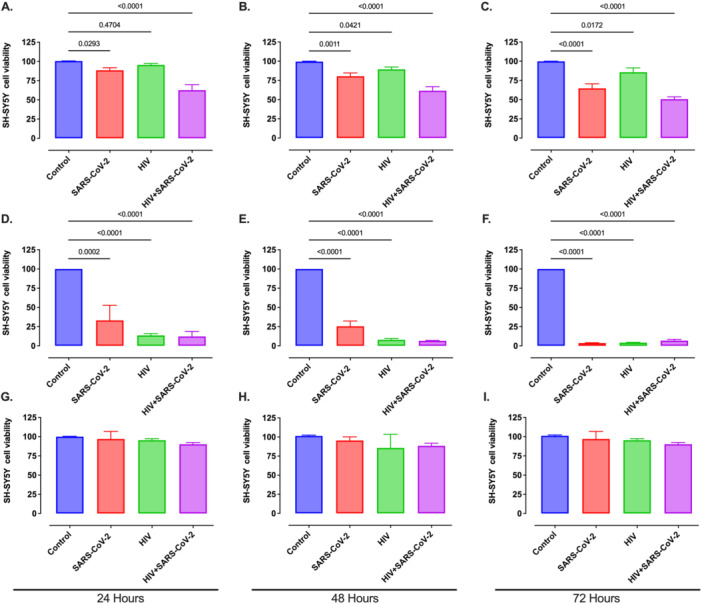
Neuronal toxicity of culture supernatant from SARS‐CoV‐2 infected astrocytes, pericytes, and microglia in presence and absence of HIV‐1 infection. Differentiated SH‐SY5Y cells were treated with culture supernatant collected from all the 4 groups as described in Figure 1 at different time points and cell viability was measured by MTT assay. (A–C) Astrocytes culture supernatant at 24, 48, and 72 hpi; (D–F) Pericytes culture supernatant at 24, 48, and 72 hpi; and (G–I) Microglia culture supernatant at 24, 48, and 72 hpi. The astrocytes and pericytes from three independent donors were included in this study. Microglia (C20) cells were tested in triplicates. All HIV/SARA‐CoV‐2 infection experiments were performed in triplicates. SH‐SY5Y cells were treated with culture supernatant in 4 replicates for cell viability assay. A one‐way analysis of variance (ANOVA) was conducted to examine the effect of conditioning media on SH‐SY5Y cells viability. Tukey's multiple comparisons test was performed to identify pairwise differences between the groups, and statistical significance *p* < 0.05 was considered significant.

Graph Pad Prism 11.0 software was used to carry out statistical analysis and plot the Figures 2, 3, Supplementary Figure 1, and Supplementary Figure 2. To compare differences between fold increase in SARS‐CoV‐2 viral loads in mono vs HIV coinfected astrocytes, pericytes, and microglia at 24 h, 48 h, and 72 h post infection, multiple unpaired t‐tests were performed. *p*‐values were adjusted using the two‐stage step‐up method of Benjamini, Krieger, and Yekutieli‐Hochberg false discovery rate (FDR) procedure. Statistical significance was defined as an adjusted *p* < 0.05. To examine the effect of conditioning media from SARS‐CoV‐2 infected astrocytes, pericytes, and microglia in the presence and absence of HIV on SH‐SY5Y cells viability, a one‐way analysis of variance (ANOVA) was conducted. Tukey's multiple comparisons test was performed to identify pairwise differences between the groups. The gene expression data were analyzed using a two‐way ANOVA to evaluate the main effects of SARS‐CoV‐2 infection in the presence and absence of HIV and longitudinal time points (24, 48, 72 hpi), as well as their interaction. Where a significant interaction was detected, group means were compared using Tukey's multiple comparisons test.

## 3 Results

### 3.1 SARS‐CoV‐2 Infection in the Brain‐Derived Astrocytes, Pericytes, and Microglia in the Presence and Absence of HIV

To determine SARS‐CoV‐2 replication and the influence of HIV on its replication kinetics, we first infected astrocytes, pericytes, and microglia with HIV, as these CNS cell types are permissive to HIV [34, 35]. Our results demonstrated that astrocytes (average HIV RNA copies per mL culture: 2.29 × 10^5^), pericytes (average HIV RNA copies per mL: 3.32 × 10^5^), and microglia (average HIV RNA copies per mL: 6.24 × 10^6^) supports varying degrees of HIV replication at 7 dpi (Supplementary Figure 1). HIV RNA levels were 27‐fold and 18‐fold higher in microglia compared with astrocytes and pericytes, respectively.

We next examined SARS‐CoV‐2 replication kinetics using RT‐qPCR. The viral RNA copies at 1‐h post‐infection were considered as baseline. In SARS‐CoV‐2‐infected astrocytes, mean viral RNA increased by 2.7‐fold at 24 h, 2.8‐fold at 48 hpi, and 2.7‐fold at 72 hpi. In HIV‐1/SARS‐CoV‐2 co‐infected astrocytes, we observed a 2.8‐fold increase at 24 hpi, a 3.3‐fold increase at 48 hpi, and a 2.9‐fold increase at 72 hpi in SARS‐CoV‐2 RNA level (Figure 2A). Similarly, SARS‐CoV‐2‐infected pericytes showed a 3.8‐fold increase at 24 hpi, a 3.7‐fold at 48 hpi, and a 3.7‐fold at 72 hpi in mean viral RNA copies from the three donors. On the other hand, in HIV‐1 and SARS‐CoV‐2 co‐infected pericytes, we observed a 2.0‐fold increase in mean viral RNA at 24 hpi, a 2.1‐fold increase at 48 hpi, and a 1.97‐fold increase at 72 hpi in mean viral RNA copies (Figure 2B). However, in SARS‐CoV‐2–infected microglia, we observed a mean fold change of 18.6 at 24 hpi, 4.1 at 48 hpi, and 1.31 at 72 hpi. Similarly, in HIV/SARS‐CoV‐2 coinfected microglia, the fold changes were 22.26 at 24 hpi, 3.7 at 48 hpi, and 1.1 at 72 hpi (Figure 2C) with modestly higher SARS‐CoV‐2 infection at 24 hpi and reduced at a later stage. Taken together, astrocytes and pericytes permit restricted SARS‐CoV‐2 infection, as evidenced by minimal increases in viral RNA in the presence and absence of HIV. Distinct from this in microglia, there was a substantial higher level of SARS‐CoV‐2 viral RNA present at 24 hpi compared to astrocyte and pericytes.

### 3.2 Conditioned Media From SARS‐CoV‐2 and HIV Infected, As Well As HIV/SARS‐CoV‐2 Co‐Infected Astrocyte and Pericyte, Induce Neuronal Cell Death

We investigated whether soluble factors released in culture media from SARS‐CoV‐2, HIV, and HIV/SARS‐CoV‐2 co‐infected cells are toxic to the neuronal cells, conditioned media were collected at 0‐, 24‐, 48‐, and 72‐hpi from uninfected, SARS‐CoV‐2–infected, HIV‐infected, and HIV/SARS‐CoV‐2 coinfected astrocytes, pericytes, and microglia, and applied to differentiated SH‐SY5Y neuroblastoma cells. At the 24 h timepoint collected conditioned media from SARS‐CoV‐2 and HIV/SARS‐CoV‐2 infected astrocytes revealed a significant reduction in neuronal cell viability (Figure 3A), whereas at 48 h and 72 h timepoint collected conditioned media from SARS‐CoV‐2, HIV, and HIV/SARS‐CoV‐2 infected astrocytes were significantly toxic to the neuronal cells as shown by reduction in cell viability (Figure 3B‐C). Pericyte‐derived supernatants, however, caused a significant decrease in neuronal viability across all infection conditions, SARS‐CoV‐2, HIV, and HIV/SARS‐CoV‐2, compared to the controls at all three time points (Figure 3D–F). Taken together, these results indicate that the soluble factors released from SARS‐CoV‐2 and HIV mono or co‐infected astrocytes and pericytes can contribute to neuronal toxicity. Interestingly, treatment of SH‐SY5Y cells with conditioned media from microglia, regardless of infection status, did not significantly affect neuronal viability (Figure 3G‐I). As microglia are highly plastic, the activation state of these cells dictates the composition of their secretome and its potential for neurotoxicity. Our results indicate that, under present experimental conditions, post‐infections with SARS‐CoV‐2 in the presence and absence of HIV, microglia may not adopt a pro‐inflammatory neurotoxic phenotype. Future experiments examining SARS‐CoV‐2 infection of microglia under various activation states, in the presence and absence of HIV, will provide further insights into its neurotoxic potential.

### 3.3 HIV/SARS‐CoV‐2 Co‐Infection Induces Proteomic Signatures in CNS Astrocytes and Pericytes, Altering Signaling Pathways Associated With Neurodegeneration

In PWH, SARS‐CoV‐2 infection has been associated with neurological complications, including neurodegenerative disorders [36]. Astrocytes and pericytes are the critical components of the BBB, play key roles in maintaining CNS homeostasis, and regulate the CNS entry of neurotropic pathogens. As described in Figure 3, only astrocytes and pericytes, but not microglia, released secretomes reduces neuronal viability, highlighting these two cell types as key mediators of neurotoxicity in the context of HIV/SARS‐CoV‐2 co‐infection. Therefore, we performed quantitative proteomic analysis of SARS‐CoV‐2 infected astrocytes and pericytes in the presence and absence of HIV‐1 to understand how the altered protein expression patterns contribute to changes in signaling pathways associated with neurocognitive outcomes. Given the comparable SARS‐CoV‐2 RNA levels at 24‐, 48‐, and 72‐hpi, our proteomic analysis targeted 24 hpi to exaine early host responses.

**Figure 4:**
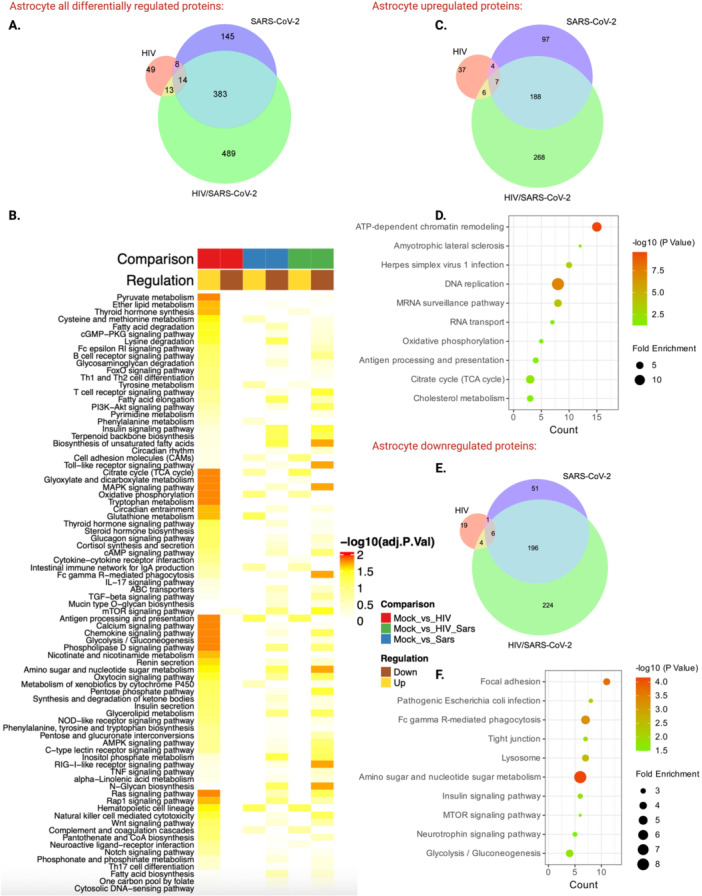
Gene‐set enrichment analysis of differentially expressed proteins in SARS‐CoV‐2 infected astrocytes in presence and absence of HIV‐1 infection. (A) Venn diagram showing all significantly regulated proteins in HIV infected, SARS‐CoV‐2 infected, and HIV/SARS‐CoV‐2 co‐infected astrocytes compared to mock infected astrocytes (p. adj < 0.05; log_2_FC ≥ 0.58); (B) Gene‐set enrichment analysis results of three pairwise comparisons, namely, Mock vs HIV, Mock vs SARS‐CoV‐2, and Mock vs HIV‐1/SARS‐CoV‐2. The heatmap visualizes negative log‐scaled adjusted *p*‐values of all up‐regulated and down‐regulated pathways; (C) Venn diagram showing significantly upregulated proteins in HIV infected, SARS‐CoV‐2 infected, and HIV/SARS‐CoV‐2 co‐infected astrocytes compared to mock infected astrocytes (p. adj < 0.05; log_2_FC ≥ 0.58); (D) Gene‐set enrichment analysis result of exclusively upregulated proteins in Mock vs HIV‐1/SARS‐CoV‐2 infected astrocytes; (E) Venn diagram showing significantly downregulated proteins in HIV infected, SARS‐CoV‐2 infected, and HIV/SARS‐CoV‐2 co‐infected astrocytes compared to mock infected astrocytes (p. adj < 0.05; log_2_FC ≥ 0.58); (F) Gene‐set enrichment analysis result of exclusively downregulated proteins in Mock vs HIV‐1/SARS‐CoV‐2 infected astrocytes. Astrocytes from three independent donors were included in this study. Signaling pathways with p. adj(dist. dir. up) and p. adj(dist. dir. dn) < 0.1 were considered significantly up‐regulated and down‐regulated respectively.

In HIV infected, SARS‐CoV‐2 infected, and HIV/SARS‐CoV‐2 co‐infected astrocytes, we detected a total of 84 (54 upregulated and 30 proteins were downregulated), 550 (296 upregulated and 254 downregulated), and 899 differentially regulated proteins (469 upregulated and 430 downregulated), respectively (Figure 4A). Interestingly, there were 268 (Figure 4C) and 224 (Figure 4E) uniquely upregulated and downregulated proteins observed in the HIV/SARS‐CoV‐2 co‐infected astrocytes. To gain insights into how the differentially expressed proteins may influence the host cellular function, we performed gene‐set enrichment analysis. A heat map of the up‐ and down‐regulated pathways in astrocytes in pairwise comparisons of mock vs HIV, mock vs SARS‐CoV‐2, and mock vs HIV/SARS‐CoV‐2 is shown in Figure 4B. In the HIV infected astrocytes, we observed several upregulated pathways, including pyruvate metabolism, TCA cycle, oxidative phosphorylation, glycolysis/gluconeogenesis, tryptophan metabolism, taurine and hypotaurine metabolism, beta‐alanine metabolism, circadian entrainment, and multiple amino acid metabolism pathways, among others. Whereas the downregulated signaling pathways in HIV infected astrocytes includes mTOR signaling pathway and Steroid biosynthesis. Similarly, in SARS‐CoV‐2‐infected astrocytes, the upregulated signaling pathways include antigen processing and presentation, glutathione metabolism, thiamine metabolism, and oxidative phosphorylation, and downregulated pathways in SARS‐CoV‐2‐infected astrocytes includes amino sugar and nucleotide sugar metabolism, biosynthesis of unsaturated fatty acids, glycerolipid metabolism, hedgehog signaling pathway, inositol phosphate metabolism, N‐Glycan biosynthesis, and sphingolipid metabolism. Moreover, in HIV/SARS‐CoV‐2 co‐infected astrocytes, we observed the upregulation of signaling pathways that include antigen processing and presentation, cell adhesion molecules (CAMs), TCA cycle, and thiamine metabolism. Whereas in HIV/SARS‐CoV‐2 co‐infected astrocytes, we observed downregulation of signaling pathways such as amino sugar and nucleotide sugar metabolism, biosynthesis of unsaturated fatty acids, C‐type lectin receptor signaling pathway, Fc gamma R‐mediated phagocytosis, glycerolipid metabolism, hedgehog signaling pathway, insulin signaling pathway, MAPK signaling pathway, N‐Glycan biosynthesis, pentose phosphate pathway, and sphingolipid metabolism among others. Further, we performed KEGG pathway analysis using the uniquely upregulated and down regulated proteins in HIV/SARS‐CoV‐2 co‐infected astrocytes to understand the impact of co‐infections in these cell types. As illustrated in Figure 4D, the uniquely upregulated proteins disrupt several pathways including ATP‐dependent chromatin remodeling, oxidative phosphorylation, TCA cycle, cholesterol metabolism, antigen processing and presentation, and HSV infection among others. Similarly, the uniquely downregulated proteins disrupt several pathways that include tight junction, lysosome, Fc gamma receptor‐mediated phagocytosis, sugar metabolism, insulin signaling pathways, mTOR signaling pathways, neurotrophins signaling pathways, and glycolysis/gluconeogenesis, as shown in Figure 4F.

**Figure 5:**
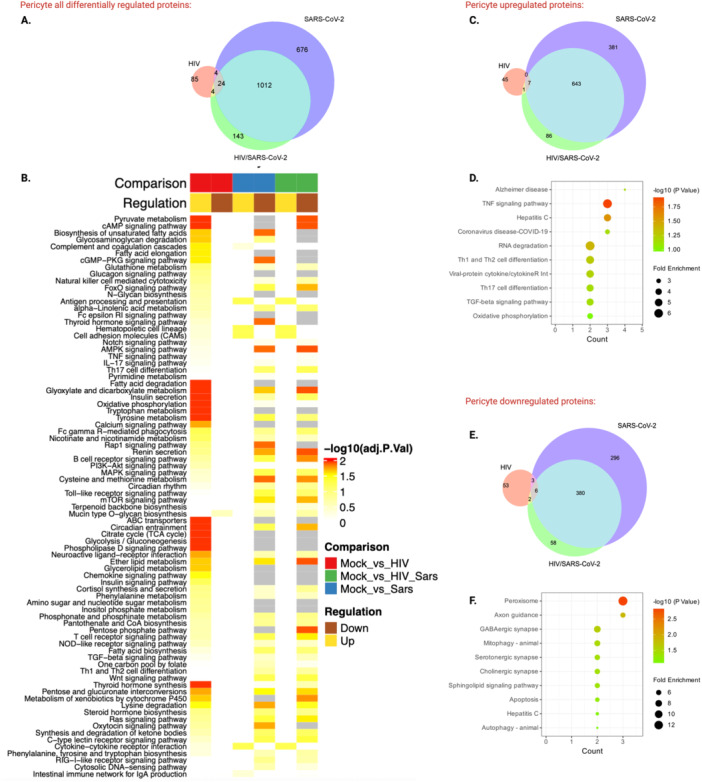
Gene‐set enrichment analysis of differentially expressed proteins in SARS‐CoV‐2 infected pericytes in presence and absence of HIV‐1 infection. (A) Venn diagram showing all significantly regulated proteins in HIV infected, SARS‐CoV‐2 infected, and HIV/SARS‐CoV‐2 co‐infected pericytes compared to mock infected pericytes (p. adj < 0.05; log_2_FC ≥ 0.58); (B) Gene‐set enrichment analysis results of three pairwise comparisons, namely, Mock vs HIV, Mock vs SARS‐CoV‐2, and Mock vs HIV‐1/SARS‐CoV‐2. The heatmap visualizes negative log‐scaled adjusted p‐values of all up‐regulated and down‐regulated pathways; (C) Venn diagram showing significantly upregulated proteins in HIV infected, SARS‐CoV‐2 infected, and HIV/SARS‐CoV‐2 co‐infected pericytes compared to mock infected pericytes (p. adj < 0.05; log_2_FC ≥ 0.58); (D) Gene‐set enrichment analysis result of exclusively upregulated proteins in Mock vs HIV‐1/SARS‐CoV‐2 infected pericytes; (E) Venn diagram showing significantly downregulated proteins in HIV infected, SARS‐CoV‐2 infected, and HIV/SARS‐CoV‐2 co‐infected pericytes compared to mock infected pericytes (p. adj < 0.05; log_2_FC ≥ 0.58); (F) Gene‐set enrichment analysis result of exclusively downregulated proteins in Mock vs HIV‐1/SARS‐CoV‐2 infected pericytes. Pericytes from three independent donors were included in this study. Signaling pathways with p. adj(dist. dir. up) and p. adj(dist. dir. dn) < 0.1 were considered significantly up‐regulated and down‐regulated respectively.

Furthermore, in HIV infected, SARS‐CoV‐2‐infected, and HIV/SARS‐CoV‐2 co‐infected pericytes, we detected a total of 117 (53 upregulated and 64 downregulated), 1716 (1031 upregulated and 685 downregulated), and 1183 differentially regulated proteins (737 upregulated and 446 downregulated), respectively (Figure 5A). Distinct from astrocytes, there were only 86 (Figure 5C) and 58 (Figure 5E) uniquely upregulated and downregulated proteins in the HIV/SARS‐CoV‐2 co‐infected pericytes, indicating that the disruption of the proteomic landscape in pericytes was driven by SARS‐CoV‐2. To gain insights into how the differentially expressed proteins may influence the host cellular function, we performed gene‐set enrichment analysis. A heat map of the up‐ and down‐regulated pathways in pericytes in pairwise comparisons of mock vs HIV, mock vs SARS‐CoV‐2, and mock vs HIV/SARS‐CoV‐2 is shown in Figure 5B. In HIV infected pericytes, we observed several upregulated pathways, including circadian entrainment, TCA cycle, oxidative phosphorylation, pyruvate metabolism, tryptophan metabolism, neuroactive ligand‐receptor interaction, glycerolipid metabolism, sphingolipid metabolism, thiamine metabolism, insulin signaling pathway, taurine and hypotaurine metabolism, and glutathione metabolism, among others. Whereas there are no downregulated signaling pathways in this group. Similarly, in SARS‐CoV‐2 infected pericytes, we observed upregulated signaling pathways such as antigen processing and presentation, cell adhesion molecules (CAMs), and ECM‐receptor interaction. Further, the downregulated signaling pathways in SARS‐CoV‐2‐infected pericytes include AMPK signaling pathway, alanine, aspartate, and glutamate metabolism, amino sugar and nucleotide sugar metabolism, arginine and proline metabolism, arginine biosynthesis, C‐type lectin receptor signaling pathway, calcium signaling pathway, circadian entrainment, and ECM‐receptor interaction. Moreover, in HIV/SARS‐CoV‐2 co‐infected pericytes, we observed the upregulation of signaling pathways that include antigen processing and presentation, cell adhesion molecules (CAMs), and ECM‐receptor interaction. Whereas, the down regulated signaling pathways in SARS‐CoV‐2 infected pericytes includes AMPK signaling pathway, alanine, aspartate and glutamate metabolism, amino sugar and nucleotide sugar metabolism, C‐type lectin receptor signaling pathway, calcium signaling pathway, circadian entrainment, TCA cycle, Fc gamma R‐mediated phagocytosis, glucagon signaling pathway, HIF‐1 signaling pathway, Histidine metabolism, Insulin signaling pathway, pentose phosphate pathway, pyruvate metabolism, sphingolipid metabolism, tryptophan metabolism, and mTOR signaling pathway among others. Further, we performed KEGG pathway analysis using the uniquely upregulated and downregulated proteins in HIV/SARS‐CoV‐2 co‐infected pericytes to understand the impact of co‐infections in these cells. As illustrated in Figure 5D, the uniquely upregulated proteins disrupt several pathways, including TNF signaling, RNA degradation, Th1/Th2 and Th17 cell differentiations, TGF‐β signaling, oxidative phosphorylation, Alzheimer's disease, and COVID‐19, among others. Similarly, the uniquely downregulated proteins disrupt several pathways that include apoptosis, autophagy, mitophagy, peroxisome, sphingolipid metabolism pathway, axon guidance, GABAergic synapse, serotonergic synapse, and cholinergic synapse, as depicted in Figure 5F.

Taken together, these results showed that SARS‐CoV‐2 infection, alone or with HIV co‐infection, induces overlapping changes in protein expression patterns in astrocytes and pericytes, highlighting potential shared functional interactions among the uniquely regulated pathways, and these proteomic signatures are associated with pathways implicated in neurological disorders.

### 3.4 Gene Expression Analysis of SARS‐CoV‐2 Entry Receptors and Inflammatory Factors in Astrocytes and Pericytes, Reflecting Possible Altered Cellular Susceptibility

We next examined the expression pattern of host cell receptors involved in SARS‐CoV‐2 entry, as well as inflammatory markers, in astrocytes and pericytes infected with SARS‐CoV‐2 in the presence and absence of HIV. Changes in the expression levels of cellular receptors and host factors that support SARS‐CoV‐2 infection, such as ACE‐2, TMPRSS2, NRP1, and TRIM28, were determined at 24, 48, and 72 hpi (Supplementary Figure 2). In SARS‐CoV‐2‐infected astrocytes, the expression of ACE2 peaks at 24 h post‐infection with a 7.4‐fold increase, followed by a gradual decrease to 3.4‐fold and 2.1‐fold change at 48 h and 72 h, respectively. Similarly, in HIV/SARS‐CoV‐2 co‐infected cells, ACE‐2 expression showed a 4.0‐fold increase at 24 h, followed by a gradual decrease to 2.7‐fold and 0.6‐fold change at 48 h and 72 h, respectively. The expression of other SARS‐CoV‐2 entry factors, like TMPRSS2, NRP1, and TRIM28, was only slightly altered. IL‐6 expression increased by 4.6‐, 4.4‐, and 2.0‐fold in SARS‐CoV‐2‐infected, and a 2.3‐, 2.4‐, and 1.6‐fold in HIV/SARS‐CoV‐2‐coinfected astrocytes at 24, 48, and 72 h. post‐infection, respectively (Supplementary Figure 2). Similarly, IL‐1β expression increased by 9.8‐, 8.6‐, and 8.6‐fold in SARS‐CoV‐2‐infected, and by 3.7‐, 5.3‐, and 3.9‐fold in HIV/SARS‐CoV‐2‐coinfected astrocytes over the same time points (Supplementary Figure 2). These data indicated that IL‐6 and IL‐1β, key mediators of inflammation, are induced in HIV, SARS‐CoV‐2, and HIV/SARS‐CoV‐2 co‐infected astrocytes. However, unlike astrocytes, in pericytes, we observed a decrease in ACE2 expression in both SARS‐CoV‐2 and HIV/SARS‐CoV‐2 coinfected cells at all time points, and a slight increase in the expression of TMPRSS2, NRP1, TRIM28, IL‐6, TNF‐α, IL‐1β, and IL‐18 in both groups at all time points (Supplementary Figure 2).

In summary, our results revealed a modest increase in the expression of SARS‐CoV‐2 host cell entry receptors and IL‐6 and IL‐1β pro‐inflammatory mediators in astrocytes, particularly at 24 hpi. The gene expression changes in pericytes were less pronounced than those observed in astrocytes.

## 4 Discussion

People exposed to SARS‐CoV‐2 suffer from neurocognitive impairments, both during acute infection and in PASC [37]. The impact of SARS‐CoV‐2 infection in the CNS of PWH, who may already have compromised BBB and persistent neuroinflammation, remains incompletely understood. To address this, we investigated the effects of HIV/SARS‐CoV‐2 co‐infection in human microglia, astrocytes, and pericytes. We observed variable levels of SARS‐CoV‐2 replication in brain‐derived cells in the presence and absence of HIV. However, SARS‐CoV‐2 mono and co‐infection with HIV resulted in an increase in the expression of viral host cell entry factors and pro‐inflammatory mediators like IL‐6 and IL‐1β in astrocytes. Additionally, the culture supernatant collected from mono or co‐infected astrocytes and pericytes was neurotoxic, resulting in a substantial increase in neuronal death compared to uninfected controls. Using quantitative proteomic analysis, we identified a distinct protein expression signature in co‐infected astrocytes/pericytes. The significantly enriched pathways in co‐infected astrocytes/pericytes are associated with the altered energy metabolism pathways, immune dysfunctions, synaptic transmission, and others associated with the development of neurological disorders such as PD, AD, HD, and ALS. Several symptoms of these diseases are reported among neuro‐PASC patients [38]. These findings indicate the importance of further studies on understanding the SARS‐CoV‐2‐mediated neurological dysfunctions among PWH, as they frequently suffer from neuro‐HIV [39].

The expression of host cell entry factors plays an essential role in establishing and persistent viral infection. We examined the expression of SARS‐CoV‐2 entry receptors in astrocytes and pericytes. Our findings align with previous studies, including ours, showing low to moderate expression of ACE2 and NRP1 in brain cells [40, 41, 42, 43]. Studies using brain tissue from COVID‐19 patients suggest that ACE2‐expressing pericytes may facilitate SARS‐CoV‐2 entry into the brain [21]. Similarly, in cortical assembled models, pericyte‐like cells expressing ACE2 serve as sites for SARS‐CoV‐2 replication [44]. Furthermore, studies on human monocyte‐derived macrophages have shown that restricted SARS‐CoV‐2 infection triggers dysregulated immune responses and overexpression of pro‐inflammatory cytokines, establishing an inflammatory microenvironment in brain tissues [45, 46]. Taken together, these findings support our observations that restrictive SARS‐CoV‐2 infection in the presence and absence of HIV in astrocytes and pericytes leads to an increase in inflammatory cytokine/chemokine release, which are neurotoxic.

Many people after recovery from acute COVID‐19 are developing cognitive impairments, and atrophy in the orbitofrontal and the para‐hippocampal regions of the brain [47, 48]. These symptoms are similar to CNS complications reported in PWH with neuro‐HIV [49]. Therefore, it is important to understand the impact of SARS‐CoV‐2 on neurological complications among PWH. Published literature demonstrates a heterogeneous distribution of SARS‐CoV‐2 genomic RNA and proteins in the brain of COVID‐19 patients even after recovery from active disease [44, 50, 51, 52, 53]. HIV status is strongly associated with the development of PASC [54], and a recent study from a cohort of PWH on suppressive ART (RV254 study) reports a significant decline in cognitive performance persisting at least 6 months post‐recovery from acute COVID‐19, suggesting long‐term neurological complications [55]. Our findings from in‐vitro culture models indicate that even in the absence of a productive infection, SARS‐CoV‐2 can induce a unique proteomic signature in the presence of HIV, associated with signaling pathways responsible for the development of cognitive decline in PWH.

Astrocytes play a critical role in maintaining BBB integrity, regulating cerebral blood flow, and water homeostasis, serving as an energy source for neurons, and maintaining the balance of glutamate‐glutamine levels [56, 57, 58]. However, during a pathogenic insult, astrocytes undergo morphological, transcriptional, and functional changes, contributing to the development of neurodegenerative disorders [59]. Additionally, studies have shown that astrocytes can act as reservoirs for HIV with restricted replication and efficient cell‐to‐cell transmission capability [60, 61]. HIV infection induces activation of astrocytes, leading to the release of pro‐inflammatory cytokines/chemokines and glutamate, causing excitotoxicity and neuronal death [62]. We observed differential modulation of several energy metabolism pathways in HIV/SARS‐CoV‐2 co‐infected astrocytes, which can potentiate HIV associated dysregulation of astrocytes. These findings suggest that PWH should be closely monitored for the development of neurodegenerative disorders after recovering from COVID‐19.

In the brain, pericytes remain embedded between brain microvascular endothelial cells, astrocytes, and neurons. Recent research suggests that pericytes can support HIV replication [35, 63], resulting in the loss of pericytes from the BBB tight junction, increasing BBB permeability, and facilitating the transmigration of peripheral leukocytes in the brain parenchyma. This also leads to overexpression of pro‐inflammatory mediators like IL‐6, TNF‐α, and IL‐1β in pericytes, which can trigger neuroinflammation [64, 65].

In pericytes, we observed downregulation of ACE2 after entry of SARS‐CoV‐2 in the presence and absence of HIV, which does not preclude intracellular changes in proteomic landscape. This observation is in line with previous reports of SARS‐CoV‐2 mediated ACE2 down‐regulation, which triggers changes in downstream gene expression associated with cytokine signaling, respiratory distress, and contribute to the severity of COVID‐19 [66]. However, the mechanism of ACE2 degradation post viral entry, as well as the role of SARS‐CoV‐2 proteins in this process, remains poorly understood [67]. In pericytes co‐infected with HIV/SARS‐CoV‐2, we observed disruptions of several signaling pathways, including energy balance, adaptive immune response, cytokine signaling, and neurotransmission linked with the development of depression [68, 69]. This finding is also supported by the fact that many COVID‐19 patients living with HIV develop depression after recovery from acute infection [70]. Mallat et al. describe the spike protein of SARS‐CoV‐2 damaging the vascular and immune regulatory functions of pericytes, resulting in vascular‐mediated brain damage [71]. We also observed several immune‐related pathways uniquely regulated in HIV/SARS‐CoV‐2 co‐infected pericytes, like the Fc gamma receptor‐mediated phagocytosis pathway.

A key limitation of this study is the inclusion of limited number of brain cell types, that includes microglia, astrocytes, and pericytes for virologic assessments, and astrocytes and pericytes for proteomic analysis. Additionally, our proteomic analysis was specially designed to identify intracellular pathways driving the release of neurotoxic secretomes rather than focusing on extracellular components such as culture supernatants; however, complementary comparative analysis is warranted. The human brain is a complex organ composed of diverse, interconnected cell populations that function as integrated network. Consequently, the overall neurological consequence of HIV/SARS‐CoV‐2 co‐infection is challenging to replicate using isolated cell types, representing a limitation of the current study [49, 72]. Furthormore, factors such as response to vaccinations, and antiviral therapies should be considered when evaluating neurological outcomes of SARS‐CoV‐2 among PWH. This limitations underscores the need for a pre‐clinical animal model to further validate and extend our findings in future studies.

## 5 Conclusions

Our findings suggest that brain‐resident cells, including microglia, astrocytes, and pericytes, can support SARS‐CoV‐2 replication in the presence and absence of HIV infection. Notably, HIV/SARS‐CoV‐2 co‐infection in the astrocytes and pericytes induces neurotoxic effects and generates a distinct proteomic signature that disrupts key cellular pathways involved in immune responses, energy metabolism, autophagy/mitophagy, and neuronal signaling. These alterations are associated with the mechanisms implicated in several neurodegenerative disorders, including PD, AD, Huntington's disease, and ALS. Collectively, these findings highlight the importance of monitoring PWH following SARS‐CoV‐2 infection for plausible development or exacerbation of neurological complications.

## Author Contributions

**Arpan Acharya:** conceptualization, methodology, data curation, formal analysis, writing original draft preparation, reviewing and editing. **Michellie Thurman:** data curation. **Debapriya Sutar:** data curation. **Oluwatayo Israel Olasunkanmi:** data curation. **Johid R. Malik:** resources and support with the establishment of neuronal cell culture methods and reviewed the manuscript. **Shetty Ravi Dyavar:** Resources and support with the establishment of neuronal cell culture methods and reviewed the manuscript. **Ákos Végvári:** data curation. **Siddappa N. Byrareddy:** conceptualization, funding acquisition, formal analysis, writing‐ reviewing and editing.

## Ethics Statement

The authors have nothing to report.

## Conflicts of Interest

The authors declare no conflicts of interest.

## Supporting information


**Figure S1:** HIV‐1 viral loads in astrocytes, pericytes, microglia. 7 days post‐infection, the cell‐free HIV‐1 RNA concentration in culture supernatant from (A) astrocytes, (B) pericytes, and (C) microglia were measured by RT‐qPCR. The astrocytes and pericytes from three independent donors were included in this study. All experiments were performed in triplicates and repeated three times. Microglia (C20) cells were tested in triplicates. A one‐way analysis of variance (ANOVA) was conducted, and Tukey's multiple comparisons test was performed to identify pairwise differences between the groups, and statistical significance was defined as *p* < 0.05.

**Figure S2:** Differential gene expression analysis of SARS‐CoV‐2 host cell entry receptors and inflammatory factors in SARS‐CoV‐2 and HIV‐1/SARS‐CoV‐2 infected astrocytes and pericytes compared to uninfected controls. SARS‐CoV‐2 and HIV‐1/SARS‐CoV‐2 infected astrocytes 24 h (A), 48 h (B), and 72 h (C) post SARS‐CoV‐2 infection. SARS‐CoV‐2 and HIV‐1/SARS‐CoV‐2 infected pericytes 24 h (D), 48 h (E), and 72 h (F) post SARS‐CoV‐2 infection. The astrocytes and pericytes from three independent donors were included in this study. All experiments were performed in triplicates and repeated three times. A two‐way ANOVA was performed to evaluate the effects of SARS‐CoV‐2 infection in presence and absence of HIV and longitudinal time points (24, 48, 72 hpi), as well as their interaction. In case of a significant interaction, group means were compared using Tukey's multiple comparisons test. Statistical significance was defined as *p* < 0.05.

## Data Availability

The data that support the findings of this study are available on request from the corresponding author. The data are not publicly available due to privacy or ethical restrictions. The mass spectrometry proteomics data have been deposited in the ProteomeXchange Consortium via the PRIDE partner repository (PXD043206). The rest of the data generated in this study are included in this manuscript.
